# Early recombinant human growth hormone treatment improves mental development and alleviates deterioration of motor function in infants and young children with Prader–Willi syndrome

**DOI:** 10.1007/s12519-022-00653-y

**Published:** 2022-12-24

**Authors:** Ruo-Qian Cheng, Yan-Qin Ying, Zheng-Qing Qiu, Jun-Fen Fu, Chun-Xiu Gong, Yan-Ling Yang, Wei Shi, Hui Li, Ming-Sheng Ma, Chang-Yan Wang, Min Liu, Jia-Jia Chen, Chang Su, Xiao-Ping Luo, Fei-Hong Luo, Wei Lu

**Affiliations:** 1 Department of Endocrinology and Inherited Metabolic Diseases, National Children’s Medical Center, Children’s Hospital of Fudan University, 399 Wan Yuan Road, Shanghai, 201102 China; 2 Department of Pediatrics, Tongji Hospital, Tongji Medical College, Huazhong University of Science and Technology, Wuhan, China; 3 Department of Pediatrics, Peking Union Medical College Hospital, Chinese Academy of Medical Sciences and Peking Union Medical College, Beijing, China; 4 Department of Endocrinology, Children’s Hospital, Zhejiang University School of Medicine, Hangzhou, China; 5 Department of Endocrinology, Genetics and Metabolism, Beijing Children’s Hospital, Capital Medical University, Beijing, China; 6 Department of Pediatrics, Peking University First Hospital, Beijing, China; 7 Department of Rehabilitation, National Children’s Medical Center, Children’s Hospital of Fudan University, Shanghai, China

**Keywords:** Body mass index, Growth hormone, Mental development, Motor development, Prader–Willi syndrome

## Abstract

**Background:**

Recombinant human growth hormone (rhGH) therapy has shown to improve height and body composition in children with Prader–Willi syndrome (PWS), the evidence of early rhGH treatment on motor and mental development is still accumulating. This study explored the time effect on psychomotor development, anthropometric indexes, and safety for infants and young children with PWS.

**Methods:**

A phase 3, single-arm, multicenter, self-controlled study was conducted in six sites. Patients received rhGH at 0.5 mg/m^2^/day for first four weeks, and 1 mg/m^2^/day thereafter for up to 52 weeks. Motor development was measured using Peabody Developmental Motor Scales-second edition, mental development using Griffiths Development Scales-Chinese (GDS-C). Height standard deviation score (SDS), body weight SDS, and body mass index (BMI) SDS were also assessed.

**Results:**

Thirty-five patients were enrolled totally. Significant improvements were observed in height, body weight, and BMI SDS at week 52; GDS-C score showed significant improvement in general quotient (GQ) and sub-quotients. In a linear regression analysis, total motor quotient (TMQ), gross motor quotient (GMQ), and fine motor quotient were negatively correlated with age; however, treatment may attenuate deterioration of TMQ and GMQ. Changes in GQ and locomotor sub-quotient in < 9-month group were significantly higher than ≥ 9-month group. Mild to moderate severity adverse drug reactions were reported in six patients.

**Conclusion:**

Fifty-two-week treatment with rhGH improved growth, BMI, mental development, and lessened the deterioration of motor function in infants and young children with PWS. Improved mental development was more pronounced when instituted in patients < 9 months old.

**Supplementary Information:**

The online version contains supplementary material available at 10.1007/s12519-022-00653-y.

## Introduction

Prader–Willi syndrome (PWS) is a rare, complex, neurodevelopmental disorder due to lack of paternal chromosome 15q11.2-q13 expression [1, 2]. Paternal interstitial deletion of the 15q11-13 region and maternal uniparental disomy (mUPD) of chromosome 15 are the most common genetic subtypes of PWS, with imprinting defects accounting for around 1%–3% of PWS patients [1]. There are some phenotypic differences between the two largest classes of genetic subtypes. Those with mUPD appear to have a higher verbal intelligence quotient and milder behavioral problems, while psychosis and autism spectrum disorders are more common in this genotype [1].

Dysfunction involving various hypothalamic systems may predispose patients with PWS to a number of symptoms [1]. The clinical presentation of PWS occurs very early in life and this includes hypotonia, growth retardation, feeding difficulties, failure to thrive in infancy, delayed psychomotor and language development, and cognitive impairment [1]. From adolescent to adulthood, cognitive impairment usually in the form of mild mental retardation, excessive eating, and behavior problems are common features of PWS [1]. Improving the long-term prognosis of PWS patients' psychomotor development remains difficult to address and is the focus of current research.

Due to the innate nature of PWS, patients usually develop growth hormone (GH) deficiency at infancy or during the childhood period, which led to the approval of recombinant human GH (rhGH) in PWS patients [2]. It is widely accepted that rhGH replacement therapy has many benefits in terms of improving growth, body composition, and even health-related quality of life [2–8]. However, an important aspect of rhGH treatment pertains to improvement in mental development [1, 9]; although more than 20 manuscripts have been published, the clinical findings are not always consistent.

In infants, toddlers, and young children (aged 4–38 months in published studies), rhGH therapy could improve motor strength, mobility, and body composition [5, 10–12] through the effect on muscle thickness [13]. Several studies showed benefits both for mental and motor development assessed with different scales, including the Bayley Scales of Infant Development II (BSID-II) assessment [14, 15], Toddler and Infant Motor Evaluation (TIME) and the Capute Scales of Infant Language and Cognitive Development [6], and the Griffith test [16]. Taking all of these studies together, there is a paucity of data on the complete evaluation of the whole picture on early brain development in PWS patients. Studies that assessed both motor and mental developments including detailed descriptions of sub-development quotients for patients of different ages are still ongoing.

Children have an enhanced capacity for brain plasticity compared with adults. The brain has high plasticity during its early development through pruning of the synapses and activity-dependent refinement of neuronal connections, and many pediatric neurologic disorders have an impact on the fundamental mechanisms of brain plasticity [17]. Neuronal plasticity allows the central nervous system to learn skills and remember information and to reorganize neuronal networks in response to environmental stimulation [18]. Studies have shown that rhGH has significant neurotrophic actions in neural tissues including prosurvival effects, neuroprotection, axonal growth, synaptogenesis, neurogenesis, and neuro-re-generation [19], thus providing the rationality for early rhGH therapy in children with PWS. However, it is not clear when early treatment should commence nor its safety.

To investigate the early use of rhGH on psychomotor development beyond its physical benefits, we conducted a phase 3 study to evaluate the efficacy in terms of motor and mental development, physical improvement, and safety of daily rhGH therapy in infants and young children with PWS in China.

## Methods

### Subjects

**Fig. 1:**
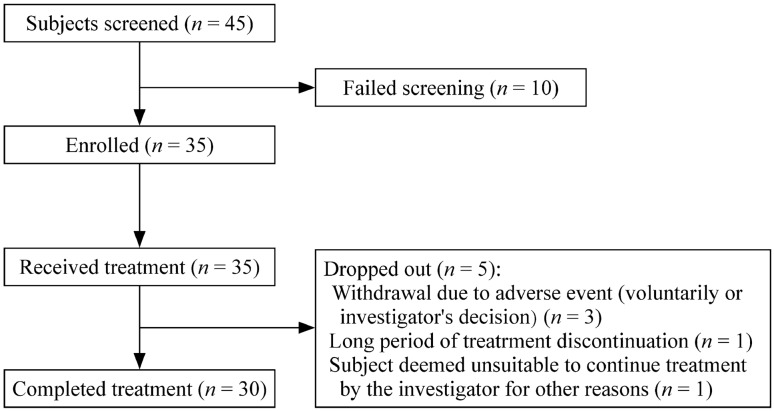
Patient enrollment flow chart of this study

A total of 45 genetically confirmed PWS subjects (42 tested using methylation-specific multiplex ligation-dependent probe amplification and three by methylation-specific polymerase chain reaction) were recruited, of whom 35 met the inclusion and exclusion criteria and received daily rhGH (Jintropin®, GeneScience Pharmaceuticals, Changchun, China) and 30 subjects completed treatment (Fig. 1). All participants fulfilled the following inclusion criteria: (1) genetically diagnosed with PWS; (2) informed consent obtained from the participant’s legal guardian; (3) agreed to complete the treatment and be assessed at scheduled visits; (4) aged between 1 month and 5 years; (5) male and female; (6) any one of the following: total motor quotient (TMQ), gross motor quotient (GMQ), or fine motor quotient (FMQ) calculated using Peabody Developmental Motor Scales-second edition (PDMS-2) less than 90 points, which corresponds to a below average score or worse [20]; (7) thyroid function is normal or maintained normal by replacement therapy; and (8) no history of GH therapy.

Exclusion criteria were: (1) abnormal liver or kidney function; (2) obvious central sleep apnea (central apnea index ≥ 5 events/hour sleep) and/or moderate or severe obstructive sleep apnea (obstructive apnea index > 5 events/hour sleep, or apnea–hypopnea index > 10 events/hour sleep), assessed by polysomnography, acute lung infection; (3) chronic diseases with long-term effects on bone metabolism and body composition; (4) congenital skeletal dysplasia, or spine scoliosis with moderate and above degree requiring treatment or claudication; (5) congenital heart disease, or an echocardiogram showing that the structural abnormalities require surgery or interventional therapy or that the left ventricular ejection fraction is < 40%, or an abnormal electrocardiogram requiring intervention; (6) history of convulsions or epilepsy; (7) other systemic chronic diseases; (8) with diagnosed tumors; (9) family history of cancers (two or more immediate family members within three generations who have had cancer), a previous history of cancer, or considered to be at a high risk of cancer after assessing other information; (10) psychosis; (11) diabetes, or abnormal fasting glucose that may affect the safety of the participant; (12) severe obesity [body mass index (BMI) above 95th percentile for the same gender and age] [21]; (13) known to be allergic to the investigational product or its excipient; (14) took part in other clinical trials within three months; (15) received drug treatment that may interfere with GH secretion or GH action within three months; and (16) other conditions that the investigator considers not suitable for enrollment into the study.

### Study design

This was a phase 3, single-arm, multicenter, self-controlled study conducted in six clinical sites in China. There was no positive control due to a lack of approved indication for rhGH treatment in PWS patients in China. A negative control was also not included due to ethical reasons. All eligible subjects were given a low dose of daily subcutaneous injection of rhGH 0.5 mg/m^2^/day for the first four weeks, then increased to 1 mg/m^2^/day thereafter for up to 52 weeks. All subjects were followed up at baseline and weeks 4, 13, 26, 39, and 52. The sample size was determined to be 30 after considering the rarity of PWS in China and a dropout rate of 20%. Before the start of the study, the study protocol, informed consent form, case report form, investigator’s manual, and other related documents were approved by the Medical Ethics Committee of the Children's Hospital of Fudan University. The study was conducted in compliance with the ethical guidelines of the Declaration of Helsinki and Good Clinical Practices and was approved by the institutional review board of each study site. The study was registered at ClinicalTrials.gov, identifier NCT03554031.

### Outcomes and assessments

The primary objective of the study was to evaluate the effectiveness of rhGH treatment on motor development. The secondary objectives were to assess the effectiveness of rhGH treatment in terms of mental development, growth, and BMI.

#### Motor and mental development assessments

Motor and mental development of the subjects were measured using PDMS-2 [20, 22] and the Griffiths Development Scales-Chinese (GDS-C) [23], respectively, at baseline and weeks 26 and 52. These assessments were conducted by professionally trained and qualified assessors. The primary outcome measure was change in (Δ) TMQ calculated according to PDMS-2 with treatment; ΔGMQ and ΔFMQ were also assessed as secondary outcome measures. The general quotient (GQ) of the GDS-C contains six sub-quotients: locomotor quotient (AQ), personal-social quotient (BQ), language quotient (CQ), eye and hand co-ordination quotient (DQ), performance quotient (EQ), and practical reasoning quotient (FQ). Other exploratory outcome measures were changes in the PDMS-2 subtests for stationary, locomotion, grasping, and visual–motor integration expressed using the standard score.

#### Anthropometric assessments

Throughout the study, height and weight were measured by a designated assessor at each clinical site using a designated weighing scale and height-measuring device. The average height, weight, and BMI data were converted to height standard deviation score (HT-SDS), body weight SDS (BW SDS), and BMI SDS according to the 2009 edition of Chinese growth standards adjusting for age and gender [21]. Bone age radiography was performed at baseline and week 52 at each center and these were collated and analyzed at the Children's Hospital of Fudan University by a qualified radiologist using the Tanner-Whitehouse 3 method. ΔHT-SDS, ΔBW SDS, ΔBMI SDS, and bone maturation (bone age/chronological age) were included as secondary outcome measures.

#### Biochemical growth marker assessments

At each follow-up, blood samples were collected and measured for serum insulin-like growth factor 1 (IGF-1) and IGF-binding protein 3 (IGFBP-3) levels at a central laboratory. Both serum IGF-1 and IGFBP-3 were measured by enzyme-labeled chemiluminescent immunometric assays (IMMULITE 2000; Siemens Healthcare Diagnostics Products Limited). IGF-1 SDS was calculated after adjusting for age and gender according to the reference published by Isojima et al. [24]. The IGF-1/IGFBP-3 molar ratio was calculated according to the formula described by Friedrich et al.: [IGF-1 (ng/mL) × 0.13]/[IGFBP-3 (ng/mL) × 0.03478] [25].

#### Safety assessments

Safety was monitored and assessed throughout the study duration. Adverse events (AEs) were evaluated based on clinical symptoms, vital signs, physical examination, and laboratory examination and, thereafter, coded using the Medical Dictionary for Regulatory Activities Chinese version 23.1. Antidrug and neutralizing antibodies at baseline, week 26, and week 52 were monitored.

### Statistical analysis

All statistical analyses were performed using SAS version 9.4 (SAS Institute, Cary, NC, USA). *P* < 0.05 was considered statistically significant. All quantitative variables were expressed as mean ± SD unless otherwise stated. All qualitative variables (e.g., safety parameters) were presented in frequency and percentage based on the total number of people in the analysis set. Within-group comparisons were assessed using the paired *t* test and Wilcoxon signed-rank test.

The full analysis set (FAS) comprised all cases enrolled in the study who received the experimental drug at least once and had at least one post-drug assessment evaluated according to the intention-to-treat principle. All missing TMQ data in the FAS were imputed using the last-observation-carried forward method. The per-protocol set (PPS) was a subset of the FAS excluding subjects with major protocol violations. Safety data analyses were performed on a safety set (SS) that included all subjects who had received the study drug at least once and had post-drug safety evaluation data. Week 52 was the main evaluation time point, and all effects were compared before and after treatment within the group. Study results were further stratified by genetic subtype and age of rhGH initiation. The 9-month distinction for age of rhGH initiation was selected as it corresponds to the median age where children with PWS enter another nutritional phase [26]. All exploratory analyses were carried out on the FAS. The number of subjects included in the FAS, PPS, and SS was 35 (100.0%), 29 (82.9%), and 35 (100.0%), respectively. Trends of TMQ, GMQ, and FMQ with age were calculated using PDMS-2. A linear regression model was established to explore the relationship between TMQ, GMQ, or FMQ and age, without (baseline scores) and with treatment.

## Results

### Subject baseline characteristics and demographics

A total of 16 (45.7%) male and 19 (54.3%) female subjects received treatment; 22 (63.9%) had paternal deletion, 10 (28.6%) had aberrant methylation (mUPD and imprinting defect), and three with unknown genotype. The three patients with unknown genotype were diagnosed with PWS using methylation-specific polymerase chain reaction; however, specific genetic aberrations were not distinguishable with this method. There were 20 (57.1%) and 15 (42.9%) subjects in the < 9 months and ≥ 9 months groups, respectively; statistical significances between the < 9 months and ≥ 9 months groups were observed in chronological age at baseline, chronological age at rhGH initiation, height, weight, head circumference, BMI, TMQ, FMQ, and GMQ (Table 1).

**Table 1:** Patient demographics and baseline characteristics of the full analysis set

Variables	Genetic subtype		Age at rhGH start		All (*n* = 35)
	Paternal deletion (*n* = 22)	Aberrant methylation (*n* = 10)	< 9 mon (*n* = 20)	≥ 9 mon (*n* = 15)	
Chronological age at baseline (mon)					
Mean ± SD	11.30 ± 11.18	14.30 ± 13.85	4.60 ± 1.54	25.70 ± 11.23^‡^	13.70 ± 12.88
Median (range^a^)	6.0 (3–39)	7.5 (3–39)	4.0 (3–8)	29.0 (10–44)	7.0 (3–44)
Chronological age at rhGH start (mon)					
Mean ± SD	11.50 ± 11.28	14.90 ± 14.15	4.80 ± 1.67	26.30 ± 11.25^‡^	14.00 ± 13.06
Median (range^a^)	6.0 (3–39)	8.0 (3–40)	4.5 (3–8)	29.0 (10–45)	7.0 (3–45)
Gender					
Male	10 (45.5)	4 (40.0)	9 (45.0)	7 (46.7)	16 (45.7)
Female	12 (54.5)	6 (60.0)	11 (55.0)	8 (53.3)	19 (54.3)
Gestational age (wk)	38.41 ± 2.47	39.03 ± 2.14	38.14 ± 2.32	39.31 ± 2.05^*^	38.64 ± 2.25
Birth height (cm)	48.32 ± 1.64	49.05 ± 4.56	48.93 ± 2.53	48.33 ± 3.04	48.67 ± 2.73
Birth weight (kg)	2.65 ± 0.36	2.88 ± 0.58	2.72 ± 0.45	2.76 ± 0.41	2.74 ± 0.43
Height (cm)	69.12 ± 12.41	72.75 ± 12.05	62.40 ± 3.87	83.59 ± 10.12^‡^	71.48 ± 12.80
Weight (kg)	7.40 ± 3.59	9.26 ± 4.44	5.60 ± 1.11	11.99 ± 3.58^‡^	8.33 ± 4.03
Head circumference (cm)	40.85 ± 3.23	42.39 ± 3.40	39.16 ± 1.76	45.17 ± 1.57^‡^	41.73 ± 3.44
BMI (kg/m^2^)	14.62 ± 1.65	16.51 ± 2.80	14.22 ± 1.42	16.80 ± 2.11^‡^	15.32 ± 2.16
TMQ	74.80 ± 8.75	72.80 ± 6.25	77.20 ± 7.66	68.90 ± 7.73^*^	73.60 ± 8.64
FMQ	82.40 ± 9.27	79.90 ± 5.49	84.00 ± 8.50	77.60 ± 7.16^*^	81.20 ± 8.46
GMQ	73.30 ± 8.63	71.70 ± 7.56	75.90 ± 7.35	67.20 ± 8.62^*^	72.20 ± 8.94

Data are presented as mean ± SD or as *n* (%). Chronological age is also expressed as median (range). *rhGH* recombinant human growth hormone, *BMI* body mass index, *FMQ* fine motor quotient, *GMQ* gross motor quotient, *TMQ* total motor quotient, *SD* standard deviation. ^a^Minimum and maximum values. Statistically significant compared with the < 9-month group, ^*^P < 0.05, ^‡^*P* < 0.001

### Effect of rhGH treatment on motor and mental development

**Fig. 2:**
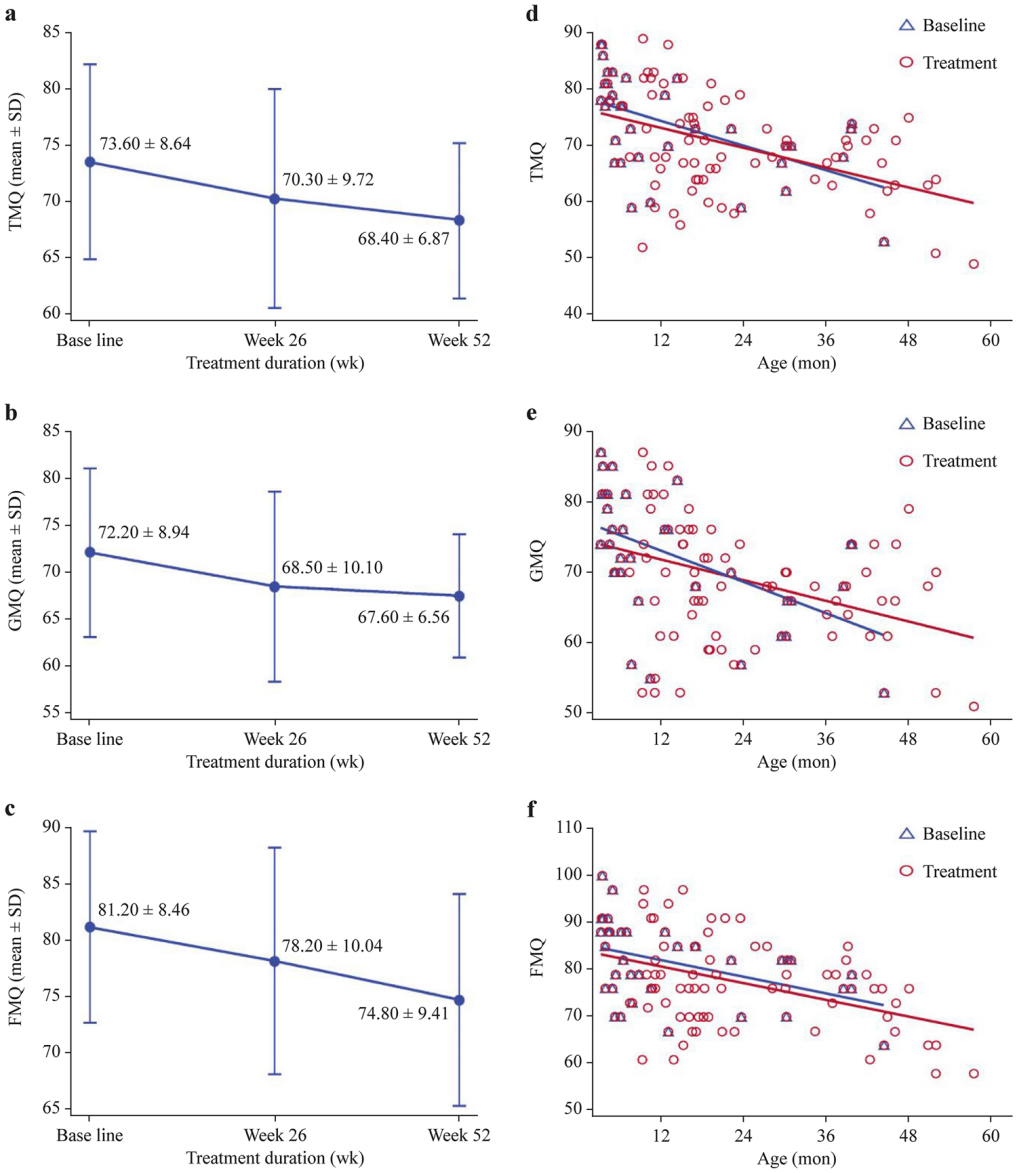
Assessment of motor development with Peabody Developmental Motor Scale-2. TMQ (**a**), GMQ (**b**), FMQ (**c**), and scatter plot and linear regression model of TMQ (**d**), GMQ (**e**), and FMQ (**f**) with and without treatment. Linear regression equation for TMQ is *y* =  − 0.3636*x* + 78.78 for baseline, and *y* =  − 0.29376*x* + 76.73 for treatment, respectively. Linear regression equation for GMQ is *y* =  − 0.3668*x* + 77.40 for baseline, and *y* =  − 0.2435*x* + 74.69 for treatment, respectively. Linear regression equation for FMQ is *y* =  − 0.2923*x* + 85.39 for baseline, and *y* =  − 0.2939*x* + 84.25 for treatment, respectively. *FMQ* fine motor quotient, *GMQ* gross motor quotient, *TMQ* total motor quotient, *SD* standard deviation

The mean TMQ decreased across 52 weeks of treatment (Fig. 2a), the ΔTMQ from baseline to week 52 was – 5.20 ± 6.88 (*P* < 0.001) (Table 2). Similarly, both mean GMQ and FMQ declined despite rhGH treatment over time (Fig. 2b, c). At week 52, statistically significant differences in ΔGMQ (− 3.90 ± 7.90, *P* = 0.015) and ΔFMQ (− 6.60 ± 8.57, *P* < 0.001) from baseline were observed (Table 2). The linear regression analysis showed that TMQ, GMQ, and FMQ were negatively correlated with age (Fig. 2d–f), meaning that these motor scores declined and the gap when compared with normal children widened as age increased (average motor quotient standard score 100). However, the linear regression slopes of TMQ and GMQ were flatter with treatment compared with those without treatment (baseline scores), indicating that the rates of decline were slower after rhGH treatment (Fig. 2d, e), narrowing the gap with normal children.

**Table 2:** Changes in motor and mental development at week 52 from baseline

Variables	All (*n* = 35)	Genetic subtype		Age at rhGH start	
		Paternal deletion (*n* = 22)	Aberrant methylation (*n* = 10)	< 9 mon (*n* = 20)	≥ 9 mon (*n* = 15)
ΔTMQ	– 5.20 ± 6.88^‡^	– 5.90 ± 7.59^*^	– 4.40 ± 5.62^*^	– 6.90 ± 7.25^‡^	– 3.00 ± 5.89
ΔGMQ	– 3.90 ± 7.90^*^	– 5.00 ± 8.03^*^	– 1.60 ± 6.46	– 6.10 ± 7.43^*^	– 0.90 ± 7.79
ΔFMQ	– 6.60 ± 8.57^‡^	– 6.30 ± 9.89^*^	– 8.60 ± 6.30^*^	– 7.20 ± 9.49^*^	– 5.80 ± 7.50^*^
ΔGQ	14.10 ± 16.80^‡^	16.70 ± 13.56^‡^	12.90 ± 24.49	24.00 ± 14.59^‡^	1.20 ± 8.81
ΔAQ	26.60 ± 20.94^‡^	29.40 ± 18.75^‡^	28.10 ± 25.19^*^	41.80 ± 10.30^‡^	6.80 ± 12.91
ΔBQ	10.9 ± 22.73^*^	16.50 ± 21.44^*^	1.40 ± 26.85	18.80 ± 23.74^*^	0.50 ± 17.13
ΔCQ	8.90 ± 22.40^*^	8.30 ± 21.77	15.00 ± 27.13	14.60 ± 27.81^*^	1.40 ± 8.64
ΔDQ	8.70 ± 22.56^*^	10.20 ± 19.88^*^	10.10 ± 31.36	16.20 ± 26.56^*^	– 1.00 ± 10.38
ΔEQ	17.20 ± 22.04^‡^	21.40 ± 20.18^‡^	10.50 ± 28.83	28.50 ± 21.18^‡^	2.50 ± 12.69

Data are presented as mean ± standard deviation. *AQ* locomotor quotient, *BQ* personal-social quotient, *CQ* language quotient, *DQ* eye and hand co-ordination quotient, *EQ* performance quotient, *FMQ* fine motor quotient, *GMQ* gross motor quotient, *GQ* general quotient, *TMQ* total motor quotient, *rhGH* recombinant human growth hormone. Statistically significant compared with baseline, ^*^P < 0.05, ^‡^*P* < 0.001. Δ change in

Among the PDMS-2 subtest standard scores, there was significant improvement in stationary subtest standard score at week 52 from baseline (1.90 ± 3.09, *P* = 0.003). Significant declines in locomotion subtest standard score (− 1.90 ± 2.93, *P* = 0.001) and visual–motor integration score (− 1.30 ± 1.70, *P* < 0.001) were observed from baseline at week 52. Grasping score also decreased from baseline to week 52, albeit not significant (− 0.90 ± 2.68, *P* = 0.080).

**Fig. 3:**
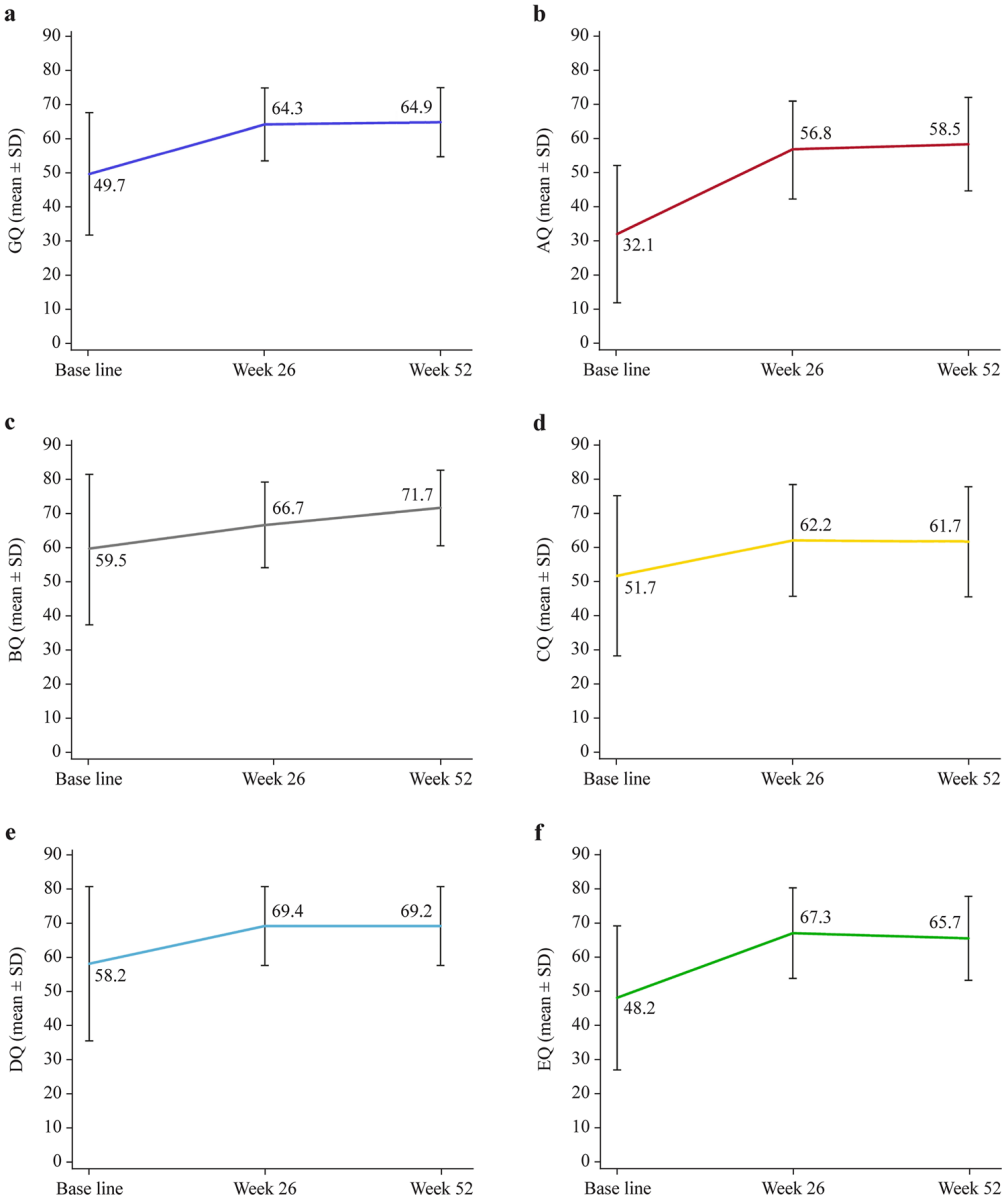
Changes in the general and sub-quotients of the Griffiths Mental Development Scale-Chinese (GDS-C). GQ (**a**), AQ (**b**), BQ (**c**), CQ (**d**), DQ (**e**), and EQ (**f**) calculated by GDS-C of the FAS. *AQ* locomotion quotient, *BQ* personal-social quotient, *CQ* language quotient, *DQ* eye and hand co-ordination quotient, *EQ* performance quotient, *GQ* general quotient*, FAS* full analysis set, *SD* standard deviation

GQ and all sub-quotients of the GDS-C showed increments from baseline to week 52 (Fig. 3a–f). At week 52, significant ΔGQ (14.10 ± 16.80, *P* < 0.001), ΔAQ (26.60 ± 20.94, *P* < 0.001), ΔBQ (10.90 ± 22.73, *P* = 0.014), ΔCQ (8.90 ± 22.40, *P* = 0.039), ΔDQ (8.70 ± 22.56, *P* = 0.043), and ΔEQ (17.20 ± 22.04, *P* < 0.001) from baseline were reported (Table 2). Statistical analyses were not computed for FQ and ΔFQ, as data were only available for one subject.

### Effect of rhGH treatment on anthropometric parameters of growth

Growth parameters are shown in Table 3. HT-SDS increased with treatment from − 1.23 ± 1.02 at baseline to 0.24 ± 0.99 at week 52 and the ΔHT-SDS was 1.50 ± 0.71 after 52 weeks of treatment (*P* < 0.001). Mean BW SDS increased from − 1.52 ± 1.46 at baseline to 0.21 ± 1.48 at week 52 and ΔBW SDS was 1.68 ± 1.45 after 52 weeks of treatment (*P* < 0.001). BMI SDS increased from − 1.20 ± 2.00 at baseline to − 0.14 ± 1.62 at week 52 and a statistically significant increment in ΔBMI SDS was observed after 52 weeks of treatment (0.95 ± 1.62, *P* = 0.003). Bone age/chronological age at week 52 was 0.98 ± 0.24, with marginal difference in bone maturity at week 52 compared with baseline (0.34 ± 0.05, *P* = 0.060).

**Table 3:** Growth parameters stratified by genetic subtype and age at rhGH start of the full analysis set

Variables	All (*n* = 35)	Genetic subtype		Age at rhGH start	
		Paternal deletion (*n* = 22)	Aberrant methylation (*n* = 10)	< 9 mon (*n* = 20)	≥ 9 mon (*n* = 15)
Baseline					
HT-SDS	– 1.23 ± 1.02	– 1.31 ± 1.02	– 0.89 ± 0.88	– 1.14 ± 1.05	– 1.35 ± 0.99
BW SDS	– 1.52 ± 1.46	– 1.97 ± 1.40^*^	– 0.72 ± 1.30^*^	– 2.29 ± 1.01^†^	– 0.49 ± 1.36^†^
BMI SDS	– 1.20 ± 2.00	– 1.85 ± 1.74^*^	– 0.28 ± 2.24^*^	– 2.46 ± 1.15^†^	0.48 ± 1.61^†^
Week 52					
HT-SDS	0.24 ± 0.99	0.23 ± 0.82	0.49 ± 1.41	0.41 ± 1.16	0.02 ± 0.70
BW SDS	0.21 ± 1.48	0.01 ± 1.40	0.68 ± 1.92	– 0.25 ± 1.49	0.81 ± 1.29
BMI SDS	– 0.14 ± 1.62	– 0.37 ± 1.62	0.15 ± 1.90	– 0.89 ± 1.35	0.85 ± 1.42
Changes at week 52 from baseline					
ΔHT-SDS	1.50 ± 0.71^‡^	1.52 ± 0.68^‡^	1.46 ± 0.87^‡^	1.51 ± 0.87^‡^	1.48 ± 0.46^‡^
ΔBW SDS	1.68 ± 1.45^‡^	1.90 ± 1.54^‡^	1.40 ± 1.35^‡^	1.96 ± 1.45^‡^	1.31 ± 1.42^‡^
ΔBMI SDS	0.95 ± 1.62^‡^	1.33 ± 1.77^‡^	0.36 ± 1.21	1.46 ± 1.42^‡^	0.29 ± 1.68

*HT-SDS* height standard deviation score, *BW SDS* body weight standard deviation score, *BMI SDS* body mass index standard deviation score, *rhGH* recombinant human growth hormone. ^*^Statistically significant comparing aberrant methylation with paternal deletion, *P* < 0.05; ^†^statistically significant comparing < 9 months with ≥ 9 months, *P* < 0.05; ^‡^statistically significant compared with baseline, *P* < 0.05. Δ change in

### Effect of rhGH treatment on biochemical growth markers

IGF-1 SDS, IGF-1/IGFBP-3 molar ratio, and serum IGFBP-3 increased from baseline to week 39 and remained stable until the end of the study. At week 52, ΔIGF-1 SDS (3.52 ± 1.64, *P* < 0.001), ΔIGF-1/IGFBP-3 molar ratio (0.11 ± 0.07, *P* < 0.001), and ΔIGFBP-3 (2211.70 ± 1012.51 ng/mL, *P* < 0.001) were statistically significant from baseline.

### Effect of rhGH treatment on mental, motor, and growth outcomes stratified by genetic subtype and age of rhGH initiation

GMQ did not decline significantly from baseline for subjects with aberrant methylation genotype. ΔGQ and all sub-quotients of the GDS-C achieved significant or non-significant increments at week 52 apart from ΔBQ in subjects with aberrant methylation genotype (Table 2). In terms of growth outcomes, only the paternal deletion group had a significant increase in BMI SDS after 52 weeks of treatment (Table 3). When stratified according to the age at which rhGH treatment was initiated, TMQ and GMQ in the ≥ 9-month group did not decline significantly at week 52 from baseline. It was only in the < 9-month group that GQ and all sub-quotients of the GDS-C increased significantly (Table 2). BMI SDS increased significantly only in the < 9-month group after 52 weeks of treatment (Table 3). After adjusting for baseline measurements and age of rhGH treatment initiation, there were no significant differences in all the motor, mental, and growth indicators between genotype subgroups. After adjusting for baseline measurements, ΔGQ and ΔAQ in the < 9-month group were significantly higher than the ≥ 9-month group after 52 weeks of treatment. The least squares mean difference between the < 9-month and ≥ 9-month groups for ΔGQ and ΔAQ was 11.08 [95% confidence interval (CI) = 3.32–18.83] and 27.95 (95% CI = 11.07–44.84), respectively.

### Safety

A total of 34 (97.1%) subjects experienced treatment-emergent AEs (TEAEs). Serious AEs (SAEs) were reported in seven (20.0%) subjects including infectious pneumonia, sepsis, hypoglycemia, seizures, and respiratory failure. No severe TEAEs or SAEs were related to the use of the study drug (Table 4). SAEs resulted in the death of one (2.9%) subject due to sepsis and was deemed unrelated to treatment; all the other SAEs were resolved. Treatment-related AEs of mild-to-moderate severity were reported in six (17.1%) subjects; one subject who experienced sleep apnea withdrew from the study. No significant changes were found in the clinical laboratory safety parameters with treatment.

**Table 4:** Adverse events of the safety set

Variables	Treatment group (*n* = 35)
Total TEAEs	34 (97.1)
TEAEs in ≥ 10% of subjects	
Upper respiratory tract infection	19 (54.3)
Fever	18 (51.4)
Pneumonia	8 (22.9)
Febrile infection	5 (14.3)
Diarrhea	5 (14.3)
Adenoidal hypertrophy	5 (14.3)
Hypoglycemia	5 (14.3)
Sleep apnea syndrome	4 (11.4)
Total TRAEs	6 (17.1)
Elevated serum alkaline phosphatase	3 (8.6)
Sleep apnea syndrome	3 (8.6)
Adenoidal hypertrophy	3 (8.6)
Elevated serum thyroid stimulating hormone	1 (2.9)
Hypothyroidism	1 (2.9)
Hypertension	1 (2.9)
SAEs	7 (20.0)
Pneumonia	4 (11.4)
Sepsis	1 (2.9)
Hypoglycemia	1 (2.9)
Seizure	1 (2.9)
Respiratory failure	1 (2.9)
Treatment withdrawal due to TEAEs	5 (14.3)
Treatment withdrawal due to TRAEs	1 (2.9)
Sleep apnea syndrome	1 (2.9)
Death due to TEAEs	1 (2.9)

Data are presented as *n* (%). *TEAEs* treatment-emergent adverse events, *TRAEs* treatment-related adverse events, *SAEs* serious adverse events

## Discussion

To our knowledge, this is the first study in China to describe the time effect of early rhGH treatment in infants and young children with PWS. After 52 weeks of treatment, besides significant improvement in anthropometric parameters, we found that PWS patients’ motor development quotients were negatively correlated with age when analyzed by linear regression models, and rhGH treatment slowed down the rate of deterioration in TMQ and GMQ. Mental development assessed by the GDS-C showed significant improvement both in GQ and AQ–EQ after treatment especially in subjects aged < 9 months.

We found that TMQ, GMQ, and FMQ were negatively correlated with age in pediatric patients with PWS in a linear regression analysis. However, treatment alleviated the deterioration of TMQ and GMQ. Several studies reported improvement in motor development scores assessed using various motor scales with rhGH treatment among PWS patients [5–7, 9, 13, 14]. In one study, rhGH treatment in infants and toddlers aged 4–37 months resulted in a positive trend of mobility and stability when assessed using TIME [6]. The age of independent walking was also earlier than typical for this condition [6]. Another study assessing motor changes in infants with a mean age of 15.5 months using TIME also reported an improvement in mobility and stability by 40.8% ± 31.0% and 48.5% ± 43.3% following six months of rhGH treatment, respectively [5]. Reus et al. also noticed a positive effect of rhGH on motor development in infants with PWS when assessed using the Alberta Infant Motor Scale (AIMS) and the Gross Motor Function Measure (GMFM) but not with BSID-II [12]. The authors explained that both AIMS and GMFM focus on gross motor function while BSID-II, which also includes fine motor skills, may be less sensitive at detecting the effects of treatment. In contrast, Festen et al. reported an improvement in motor development by + 11.2% in infants and toddlers assessed using BSID-II with one year of rhGH treatment compared with – 18.5% in the untreated control group [14]. Donze et al. showed that three years of rhGH treatment assessed using BSID-II improved both mental and motor development in infants, reducing the developmental gap between PWS and healthy peers [15], and eight years of continuous treatment with rhGH starting from infancy improved cognitive functioning in terms of vocabulary and total intelligence quotient [27]. Assessment using the PDMS-2 and the BSID-II scale may yield dissimilar findings in motor development [28]. In our study, PDMS-2 was utilized to assess motor development. PDMS-2 is often used in the clinical setting (e.g., in early childhood) to assess gross and fine motor skills along the developmental trajectory, identify delays in motor skills, establish individual goals and objectives for therapy or intervention, and monitor progress [22, 29]. We found positive, but less evident, effects of rhGH therapy in our cohort; the reasons may be due to the difference in the evaluation scales used.

We also found that younger pediatric patients had a greater improvement in mental development using the GDS-C. When stratified according to age of rhGH initiation, those in the < 9-month group performed significantly better with rhGH treatment than baseline in GQ and the other sub-quotients. General and locomotor development were significantly improved in the < 9-month group compared with the ≥ 9-month group. Our results were in line with other studies that demonstrated the positive impact of GH treatment on mental functioning in children with PWS [6, 14]. Meyers et al. reported a significant improvement in language and cognitive quotients combined with improvement in head circumference after two years of rhGH treatment [6], while Festen et al. observed significant mental development using BSID-II, noting that treatment increased mental development by 9.3% compared with – 2.9% in the untreated group after one year of follow-up among children with a median age of two years [14]. A recent meta-analysis of 10 randomized controlled trials performed by Luo et al. did not find significant improvement in cognitive development with rhGH treatment in PWS children [30]. However, the authors cautioned that the assessment of cognitive function in the randomized controlled studies included in that meta-analysis was not well represented [30]. Most studies focused on general cognition and intelligence quotients, leaving out important cognitive domains, such as language, vocabulary, and memory abilities [30]. The GDS-C used in our study provided a comprehensive developmental profile across different domains from motor function to cognitive skills [22]. Thus, the findings in our cohort can be credible.

Our results were consistent with other studies showing improvements in and normalization of anthropometric parameters [6, 7, 9]. Scheermeyer et al. reported normalization of HT-SDS and weight SDS after just one year of rhGH treatment in infants and toddlers with PWS, with continued improvement in the second year [31]. In our study, we observed normalization of HT-SDS and BW SDS after 52 weeks of GH treatment regardless of genetic subtype or age of GH initiation. Depending on the age and nutritional phase, BMI SDS increases or decreases could be considered an improvement [32]. Younger patients typically start off with hypotonia and feeding difficulties up to age nine months according to their nutritional phases. This is followed by normal feeding and growth until approximately two years of age. Therefore, in our study, BMI SDS significantly increased with treatment only in the < 9-month group, whereas the change in the ≥ 9-month group was not significant, which suggested a positive effect on improving malnutrition in these younger patients. This was consistent with the age-dependent BMI SDS trends in Festen et al., which showed increasing BMI SDS with treatment despite not being statistically significant [14]. Lecka-Ambroziak et al. also showed rhGH therapy to be most effective in improving anthropometric parameters in the youngest patients before the nutritional phase of increased appetite [33]. Overall, rhGH treatment restored physical growth, and the growth-promoting effect seemed to be more obvious among those with a more severe growth deficit at baseline or who started treatment early.

Children with PWS are sensitive to GH and have high levels of IGF-1 during rhGH treatment, increasing beyond + 2 SDS. This raises concerns about the safety issues related to high IGF-1 levels, as high levels of IGF-1 have been associated with lymphoid hyperplasia and this might increase the risk of sleep apnea [2]. As such, IGF-1 levels should be monitored regularly during treatment. IGF-1/IGFBP-3 molar ratio is another useful clinical tool to monitor the rhGH dose. In the present study, the IGF-1/IGFBP-3 ratio remained stable at 0.19 ± 0.06 with rhGH treatment, similar to the study by Gaddas et al. that reported a molar ratio of 0.19 ± 0.09 in children with PWS—well within the normal range [34].

In general, daily rhGH was well tolerated in pediatric patients with PWS throughout the 52-week treatment. Safety concerns about the potential AEs of rhGH treatment in this study were mainly focused on tonsillar hypertrophy, adenoidal hypertrophy, and upper airway obstruction, which are well documented in children with PWS [1]. Previous literature also suggests that rhGH treatment may increase adenoids and enlarge tonsils, potentially resulting in airway obstruction and aggravating sleep apnea [35]. Therefore, infants or children with PWS should be assessed for any signs of upper airway obstruction and sleep apnea before commencing treatment. In our present study, only one patient withdrew from the study due to sleep apnea, which was mild in severity and deemed related to the treatment. The majority of the TEAEs in this study were mild to moderate in severity and all adverse drug reactions did not require further intervention, suggesting that the potential benefits of rhGH outweigh its risks in infants and young children with PWS.

The merit of our study was the simultaneous use of PDMS-2 and the GDS-C to comprehensively evaluate, monitor, and accurately capture the development of different motor skills and the overall development of pediatric patients with PWS, and this study enrolled younger patients with a median age of 7.0 months, in line with increasing evidence supporting the benefit of early intervention with rhGH [1, 2, 6, 9, 14, 16, 36]. However, the short duration and small number of patients may not be able to reveal the full spectrum of rhGH potency, so future studies should recruit larger sample sizes and have longer study periods to elucidate the effect of rhGH treatment on motor and mental outcomes.

In conclusion, treatment with rhGH for 52 weeks in infants and young children with PWS improved growth (height and weight), BMI, and mental development. In addition, the results of this study support the premise that initiation of treatment at early infancy (before age nine months) yielded better mental outcomes than those who started treatment later. The impact on motor development remains inconclusive, although our linear regression analysis suggested a positive effect by alleviating the deterioration of motor function in infants and young children with PWS. The present findings of this study add to the growing evidence that rhGH administration in infants and young children with PWS is well tolerated and effective, providing benefits that extend beyond physical growth when initiated early. No rhGH was approved for the treatment of PWS in China at the time of study commencement. Therefore, the results of our study will provide more evidence to guide the clinical practice of rhGH therapy in Chinese PWS patients.

## Supplementary Information

Below is the link to the electronic supplementary material.

Supplementary file 1 (DOCX 86 KB)

## Data Availability

The data that support the findings of this study are available from the corresponding author on reasonable request.
