# Supplementary material for: Early recombinant human growth hormone treatment improves mental development and alleviates deterioration of motor function in infants and young children with Prader–Willi syndrome
Source: World J Pediatr. 2022 Dec 24;19(5):438–49. doi: 10.1007/s12519-022-00653-y (PMC10149441; doi:10.1007/s12519-022-00653-y)
Supplement: Supplementary file 1 — (DOCX 86 KB) [file 12519_2022_653_MOESM1_ESM.docx]

**Supplement Table** Studies for psychomotor development assessments with PWS patients

| **Study (Year)** | **Country** | **Subjects (Age)** | **Evaluation scales** | |  |
| --- | --- | --- | --- | --- | --- |
|  |  |  | **Motor development** | **Mental development** | |
| Myers  (2007) | USA | 25 PWS infants and toddlers  (**4~37 months**) | Toddler and Infant Motor Evaluation (**TIME**)  1. mobility  2. stability | Capture Scales (**CAT/CLAMS**) of Infant Development  1. cognitive  2. language function | |
| Festen  (2008) | Netherlands | 43 PWS infants and toddlers  **(1.3~3.1 years**) | Bayley Scales of Infant Development II (**BSID II**)   - ***Motor:*** gross and fine skills - ***Mental:*** visual and auditory information processing, language development, memory, eye–hand coordination, imitation and problem solving | |  |
| Eiholzer  (2008) | Switzerland | 26 PWS infants and toddlers | **Griffith test**   - Global DQ: global development - Five subscales:   1. DQ A: locomotion  2. DQ B: personal- social development  3. DQ C: hearing and speech  4. DQ D: hand and eye  5. DQ E: cognitive performance | |  |
| Donze  (2018) | Netherlands | 63 PWS infants and toddlers | **BSID II** (suitable for children with a developmental age between 0 and 3.5 years)   - ***Motor:*** gross and fine skills - ***Mental:*** visual and auditory information processing, language development, memory, eye-hand coordination, imitation, and problem solving | |  |
| Cheng  (2022) | China  (our study) | 35 PWS infants, toddlers and young children  **(1 month~5 years**) | Peabody Developmental Motor Scales-2 (**PDMS-2**)  1. Total Motor Quotient (TMQ)  2. Gross Motor Quotient (GMQ)  3. Fine Motor Quotient (FMQ) | **Griffith test**  1. locomotor quotient (AQ)  2. personal-social quotient (BQ)  3. language quotient (CQ)  4. eye and hand co-ordination quotient (DQ)  5. performance quotient (EQ)  6. practical reasoning quotient (FQ) | |
